# Adversarial dense graph convolutional networks for single-cell classification

**DOI:** 10.1093/bioinformatics/btad043

**Published:** 2023-01-20

**Authors:** Kangwei Wang, Zhengwei Li, Zhu-Hong You, Pengyong Han, Ru Nie

**Affiliations:** School of Computer Science and Technology, China University of Mining and Technology, Xuzhou 221116, China; School of Computer Science and Technology, China University of Mining and Technology, Xuzhou 221116, China; School of Computer Science, Northwestern Polytechnical University, Xi’an 710072, China; Central Lab, Changzhi Medical College, Changzhi 046000, China; School of Computer Science and Technology, China University of Mining and Technology, Xuzhou 221116, China

## Abstract

**Motivation:**

In single-cell transcriptomics applications, effective identification of cell types in multicellular organisms and in-depth study of the relationships between genes has become one of the main goals of bioinformatics research. However, data heterogeneity and random noise pose significant difficulties for scRNA-seq data analysis.

**Results:**

We have proposed an adversarial dense graph convolutional network architecture for single-cell classification. Specifically, to enhance the representation of higher-order features and the organic combination between features, dense connectivity mechanism and attention-based feature aggregation are introduced for feature learning in convolutional neural networks. To preserve the features of the original data, we use a feature reconstruction module to assist the goal of single-cell classification. In addition, HNNVAT uses virtual adversarial training to improve the generalization and robustness. Experimental results show that our model outperforms the existing classical methods in terms of classification accuracy on benchmark datasets.

**Availability and implementation:**

The source code of HNNVAT is available at https://github.com/DisscLab/HNNVAT.

**Supplementary information:**

Supplementary data are available at *Bioinformatics* online.

## Graphical Abstract

**Graphical Abstract:**
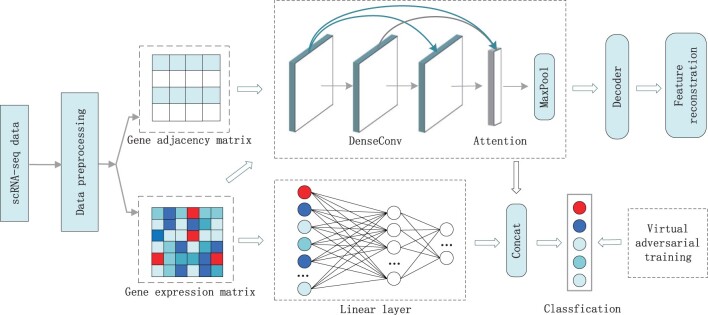

## 1 Introduction

With the rapid development of RNA-seq technology, the potential applications of single-cell transcription analysis are expanding. Single-cell transcriptomics is a crucial technique for revealing complex tissues and biological variability. Different from traditional hybridization sequencing, scRNA-seq isolates millions of cells from multiple organisms, sequences billions of gene expressions and identifies cell types based on the findings (Wilkerson *et al.*, 2021). In addition, identifying cell types is also an essential step in analyzing biological disease information and other downstream analyses (Peng *et al.*, 2020; Zheng *et al.*, 2022).

In cellular transcriptome studies, cell type prediction or other studies based on heterogeneous information and gene expression profiles are essential to elucidate stochastic biological processes. Investigating novel single-cell categorization or prediction algorithms is crucial from the biological perspective. Early studies of scRNA-seq data focused on cluster analysis to classify cells into different clusters (Plass *et al.*, 2018; Qi *et al.*, 2020), but many clustering methods require prior knowledge of multiple clusters, and the quality of clustering still needs to be improved. The key issues of clustering about single-cell are the unknown quantity of clusters, uneven cell kinds and limited scalability. In addition, the process of cluster analysis also takes a lot of time and memory (Kiselev *et al.*, 2019). To overcome these problems, more and more classification methods are being developed for the study of scRNA-seq data.

Some classification methods use predetermined similarity criteria to search for cells in the reference dataset, such as SingleR (Aran *et al.*, 2019), SCMAP (Kiselev *et al.*, 2018) and CHETAH (De Kanter *et al.*, 2019). SingleR measures the correlation by calculating Spearman correlation between gene expression. SCMAP mainly measures the maximum cell similarity between the reference and query datasets, but the calculation method for cell similarity is different. It calculates the median of a specific gene in a cell to compare the similarity with the Query cells. CHETAH correlates the input data with the reference dataset using a hierarchical classification, assigns cell types according to their correlation distribution, and also adds intermediate types of cells as transitions. However, they disregard the non-linear interactions between the labeled cells and only consider pairwise similarities. Therefore, scPred (Alquicira-Hernandez *et al.*, 2019) combines single-cell analysis techniques with machine learning methods to determine cell-specific transcriptome information for classification. scID (Boufea *et al.*, 2020) and CasTLe (Lieberman *et al.*, 2018) also apply machine learning methods to cellular transcriptome studies, mainly including logistic regression, random forest (RF), support vector machine (SVM) and k nearest neighbors (KNN). The efficacy of these methods in cellular transcriptome research has been limited despite ongoing improvements, in part because higher-order cell characteristics have not been fully utilized.

To address these challenges, cellular transcriptome research is increasingly using deep learning models. Graph Neural Network (GNN) (Rao *et al.*, 2021; Wen *et al.*, 2022; Wu *et al.*, 2020) can naturally assess low-order and high-order data features and combine them using a particular network topology. In addition, scRNA-seq data processing using GNN has been improved. scGCN (Song *et al.*, 2021) is a graph neural network method for labeling cells that applies a graph convolutional network model to single-cell histology datasets. In addition, ACTINN (Ma and Pellegrini, 2020) uses fully connected neural networks for identifying automatic cell type. GNN has unquestionably become as a valuable tool for analyzing scRNA-seq data by using convolutional kernels to extract significant information from non-Euclidean data.

However, using GNN alone may ignore low-order features of the data and lack the analysis of global data features. In addition to using single-layer convolutional neural networks, sigGCN (Wang *et al.*, 2021) attempts to combine them with fully connected neural networks. However, single-layer convolutional neural networks have limited feature learning capabilities and are more prone to overfitting issues as the network level is increased. After the number of layers increases, the local information is washed out in constant convolution and weighted average. Therefore, Fan *et al.* proposed a structured self-attention architecture (Fan *et al.*, 2020) similar to a variant of graph attention networks (GATs) (Veličković *et al.*, 2017) to aggregate different domain node features. GIN (Xu *et al.*, 2018) introduces the self-attention mechanism of aggregating the neighboring features of layer-by-layer graph nodes. We present a graph convolutional neural network based on a densely connected mechanism to address these problems. This network aggregates the information extracted from convolutional kernels of different depths using a self-attention mechanism, and fully connected neural networks are used to extract low-order features from scRNA-seq data. However, integrating multiple gene networks is equally difficult for overfitting always occurs, especially when the network level is deep, the number of cells is enormous or the total amount of input exceeds the model’s learning range. Because of this point, we use virtual adversarial training to enhance the model’s robustness, and training the neural network with noise interference can enhance the model’s ability to block interference.

Overall, this article proposes an adversarial graph convolutional networks model for single-cell classification, named HNNVAT. In our model, the filtered single-cell data is fed into a hybrid structure which consists of a fully connected network and a graph convolutional neural network. The introduction of a dense connectivity mechanism and attention-based feature aggregation improves the representation of higher-order features. To maintain the useful features of original data, a feature reconstruction module based on encoder–decoder mechanism is introduced. HNNVAT also takes advantage of virtual adversarial training to enhance robustness and generalization. On benchmark datasets, experiments demonstrate that our model outperforms the traditional methods in terms of classification accuracy. The main contributions of this article are as follows.

1. We use a gene-filtering mechanism to filter the gene with <5 expression values, and then select the gene input model providing a priori information by normalization and analysis of variance, and visualize the processing process with uniform manifold approximation and projection (UMAP).
2. We propose a hybrid neural network that not only extracts both low-order and high-order features of the data but also adaptively balances the features of the data extracted by different convolutional layers with self-attention mechanism.
3. We add noise by virtual adversarial training to simultaneously improve the robustness and generalization of the neural network.
4. The results on the benchmark datasets indicate that our model obtains better performance when compared to other methods.

## 2 Materials and methods

### 2.1 Benchmark datasets

We use six publicly available bioinformatics datasets to evaluate the performance of the new model: BaronMouse, BaronHuman, Muraro, Segerstolpe, Zhengsorted and Zheng68K. It is directly downloadable from https://doi.org/10.5281/zenodo.3357167 and increasingly used in cell annotation methods.

These datasets can be divided into two groups: Pancreatic datasets and Peripheral datasets. Specifically, Pancreatic datasets mainly include pancreatic data from the mouse and the human species. The BaronMouse dataset (Baron *et al.*, 2016) was generated by Baron *et al.* from the inDrop sequencing platform and consists of 1886 cells and 1461 genes from 13 cell populations. The remaining three pancreatic datasets were produced by Baron *et al.*, Muraro *et al.* (2016) and Segerstolpe *et al.* (2016), respectively. The BaronHuman contains 8569 cells from 14 categories and 17 499 associated genes are sequenced by the inDrop protocol. The Muraro and the Segerstolpe are divided by the CEL-Seq2 and SMARTSeq2 sequencing platforms, respectively, and they both contain approximately 2000 genes and 20 000 cells for sequencing. In addition, the Zhengsorted and Zheng68K datasets of peripheral blood mononuclear cells were sequenced using the 10× Chromium sequencing platform, and the number of sequenced genes reached 20 000 orders of magnitude. Among them, Zheng68K contains 65 943 cells and Zhengsorted contains 20 000 cells, which can be used to evaluate the generalization performance of our proposed model at different cellular levels. A brief description of the datasets is depicted in Table 1.

**Table 1.** Statistics of the six benchmark datasets

Dataset	Cells	Genes	Cell population	Sequencing platform
Zheng68K	65943	20387	11	10 × Chromium
Zhengsorted	20000	21952	10	10 × Chromium
BaronMouse	1886	1461	13	inDrop
BaronHuman	8569	17499	14	inDrop
Muraro	2122	18915	9	CEL-Seq2
Segerstolpe	2133	22757	13	SMARTSeq2

### 2.2 Data preprocessing

**Fig. 1.**
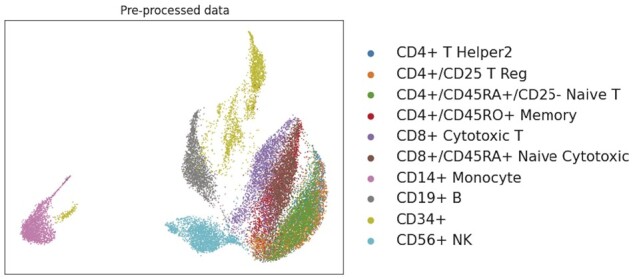
Visualization of preprocessed Zhengsorted dataset by UMAP (uniform manifold approximation and projection)

The first step in HNNVAT is data preprocessing, and it consists of two parts: cell and uncommon gene filter and feature selection. Data preprocessing determines the reliability of the data input to the model and the level of redundancy, both of which are critical for the classification outcomes of the model and the enhancement of training efficiency. Specifically, cells that are not properly labeled in the data need to be removed, mainly including cells that are unlabeled or labeled as debris and doublets. For genes, it is vital to offer adequate expression data to confirm the differences between cells and provide as little redundant information as possible. Therefore, duplicate genetic data needs to be deleted because they do not provide meaningful information about the differential expression between cells. In addition, HNNVAT filters out gene types with expression values <5 in all cells because these rare gene types can provide less valid information and are not reliable for subsequent assignment to the model. This procedure can significantly save the time spent on loading data for the model and improve the efficiency of the model operation. Next, HNNVAT performs feature selection, and the filtered gene expression values are normalized by converting them to a logarithmic scale. To select genes with high variance and maximize the extraction of available information, HNNVAT first performed ANOVA on all expression value data and ranked genes in decreasing order of expression value variance, and then selected the top 1000 genes as inputs to the model. HNNVAT carried out UMAP for the preprocessed data, as shown in Figure 1.

### 2.3 Gene adjacency matrix

Based on the outcomes of the data preprocessing, we constructed the adjacency matrix, including the top 1000 genes in each dataset. We set the diagonal of the matrix to 0, ignoring the impact of the same gene’s self-interaction. The elements on the non-diagonal are from interactions between different genes obtained through the STRING database. Thus, we constructed a weighted adjacency matrix based on the probability of gene interactions.

### 2.4 Graph convolutional network

For the single-cell classification task, the feature matrix $X\in R^{N\times M}$ input to the convolutional neural network is a matrix of gene expression values with N cells and M genes. As a weighted graph $G=\left(V,\;E,\;A\right)$, where V represents a set of nodes represented by cells whose gene expression values can be used as features of their vertices, E is a set of edges connecting the gene nodes in the graph and $A\in R^{N\times N}$ is a weighted gene adjacency matrix to represent the degree of correlation between any two genes.

The traditional Laplacian matrix is defined as L=D-A, where $D\in R^{N\times N}$ is a diagonal matrix whose values on the diagonal represent the number of edges associated with the vertex. The Laplacian matrix can be represented as $L=I+D^{-\frac{1}{2}}AD^{\frac{1}{2}}$ after normalizing, and multiplication with the feature matrix does not alter the distribution of the original cell features, where $I\in R^{N\times N}$ is a unit matrix of size N×N. The symmetric normalized Laplacian matrix also serves as the primary component of the convolution operation, which can be calculated from the eigenvalues and eigenvectors, i.e. $L=U\Lambda U^{T}$, where $U=\left(\mu_{1},\mu_{2},\ldots ,\mu_{n}\right)$, $\Lambda =\mathrm{diag}\left(\lambda_{1},\lambda_{2},\ldots ,\lambda_{n}\right)$, $\mu_{j}$ and $\lambda_{i}$ are *j*th eigenvector and *i*th eigenvalue.

The spectrogram analysis is performed in the Fourier domain through the Fourier transform $\hat{x}=U^{T}x$ and the Fourier inverse transform $x=U^{T}\hat{x}$, while the filter is represented by $g_{\theta }(\cdot )$, and the spectrogram convolution operation can be defined as $g_{\theta }*x=Ug_{\theta }\left(L\right)\;U^{T}x$, where $g_{\theta }\left(L\right)$ represents the transformed convolution kernel and x is the input of the convolution operation. Since the convolution operation in the Fourier domain consumes very expensive computational resources, one can continue to approximate the convolution kernel using the Chebyshev polynomial, i.e. $Ug_{\theta }\left(\Lambda \right)U^{T}\approx {\hat{D}}^{-\frac{1}{2}}\hat{A\;}{\hat{D}}^{\frac{1}{2}}$, where $\hat{A}=A+I$, D is the diagonal matrix, and the values on the diagonal are the summations on the corresponding rows of A. The convolution kernel operation on the final spectral map can be replaced by ${\hat{D}}^{-\frac{1}{2}}\;\hat{A}{\;\hat{D}}^{\frac{1}{2}}$, and the approximate estimation can be performed in each iteration with the already computed map filter, which saves computational resources to a large extent. The convolution calculation formula is defined as follows:

(1) $$H_{k}=\sigma \left({\hat{D}}^{-\frac{1}{2}}\hat{A}{\hat{D}}^{\frac{1}{2}}H_{k-1}W_{k-1}+b_{k-1}\right)$$

where $H_{k}$ is the neuron of the *k*th layer of convolution, $H_{k-1}$ is the input matrix of the *k*-1st layer of convolution (when k = 1, the input corresponding to the first layer of convolution is *x*), and its corresponding weight matrix is $W_{k-1}$. ${\hat{D}}^{-\frac{1}{2}}\hat{A}{\hat{D}}^{\frac{1}{2}}$ is the convolution operation calculated by the Chebyshev polynomial approximation, and $b_{k-1}$ is the bias vector of the *k*th layer, *σ* is the non-linear activation function used in the convolution layer.

### 2.5 The architecture of HNNVAT

**Fig. 2.**
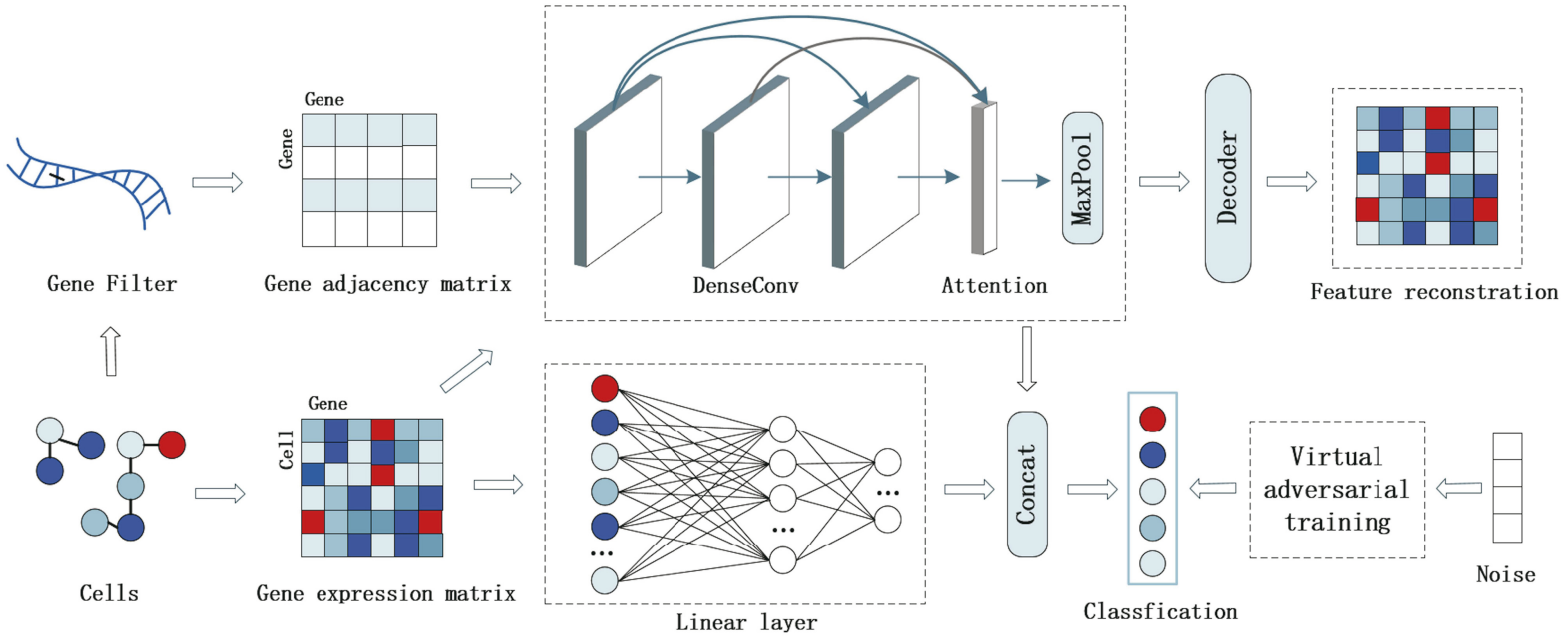
Illustration of HNNVAT for single-cell classification. HNNVAT consists of hybrid neural network, attention-based convolutional hierarchical aggregation, local feature reconstruction and virtual adversarial training

The main structure of HNNVAT is shown in Figure 2 and consists of four modules: hybrid neural network, attention-based convolutional hierarchical aggregation, local feature reconstruction and virtual adversarial training.

#### 2.5.1 Hybrid neural network

In HNNVAT, a convolutional network structure with a dense connectivity mechanism is developed to extract comprehensive cell features and expression relationships between cells and genes in different dimensions in a flexible manner. Specifically, to be able to extract node information in multiple dimensions, we input the gene expression value matrix and gene adjacency matrix into a structure with a four-layer graph convolution neural network to perform node feature extraction. However, as the extracted higher-order features tend to make the model overfit, we add direct connections between hidden layer neurons and each subsequent layer convolution, so that the information extracted from the nodes in the subsequent layers can be used to further refine the model. This enables the convolution of each layer to receive as input the structural data that was recovered from the previous convolutional layers.

Compared with convolutional neural networks, the range of features extracted by fully connected neural networks is not limited to the receptive field of convolutional kernel. Neurons in each layer are directly connected to all neurons in the next layers. To be highly complementary to the graph convolutional neural network with a dense connectivity mechanism, we use the fully connected network of the lower layers to extract global features for combination.

#### 2.5.2 Attention-based convolutional hierarchical aggregation

A serious problem of deep neural networks is that the embedding representation of nodes is prone to be over-smooth, which leads to the poor generalization ability of the single-cell classification model. To effectively utilize the information extracted from each layer of the deep convolutional network, we further use the self-attention connection mechanism between layers for information aggregation. Our HNNVAT model saves the node representation of each layer in the deep convolutional network. When the hierarchical representation information is aggregated, the corresponding attention weight is assigned to each graph-level representation to adapt to the single-cell classification task. The weight of each layer is multiplied by the output of the convolutional layer to obtain the hierarchical representation of features. It is worth noting that our model is then dimensionally transformed by the maximum pooling operation and the fully connected layer. Then, the ReLU activation function is used to enhance the non-linear expression ability of dense convolutional networks.

(2) $$H_{k}=\sigma \left(\phi \sum_{u=0}^{k-1}\alpha_{u}H_{u}W+b\right)$$

(3) $$H_{\mathrm{out}}=\sigma \left(\sum_{u=0}^{k}\alpha_{u}H_{u}\right)$$

(4) $$\alpha_{u}=\mathrm{softmax}\left(W_{2}\cdot \sigma \left(W_{1}H_{u}\right)\right)$$

where σ = ${\hat{D}}^{-\frac{1}{2}}\;\hat{A}{\;\hat{D}}^{\frac{1}{2}}$ means that the convolution operation can simplify the notation, $H_{u}$ represents the hidden layer neuron and the corresponding weight matrix W and bias vector b. $H_{\mathrm{out}}$ represents the output result of the last layer of neurons, which is an organic aggregation of all neurons, and *α* represents the attention coefficient of the corresponding neuron. Where $W_{1},\;W_{2}\in R^{N\times N}$ is the weight matrix, and softmax is used to normalize the neuron mapping results.

#### 2.5.3 Feature reconstruction

As previously mentioned, local features of various dimensions of cells and genes are obtained and aggregated after the feature information of cells is processed by the deep convolutional neural network and self-attention mechanism. However, the deep convolutional neural network will overlearn and refine graph-level features, resulting in overfitting and the model will be difficult to generalize and thus cannot accurately predict the cell categories. We design an encoder–decoder mechanism to reconstruct the node information in the feature matrix to address this issue. Specifically, a densely connected convolutional neural network and self-attention mechanism are used as encoders to encode node features into vector space. A two-layer fully connected neural network is then used as a decoder to output a reconstruction matrix of the same dimension as the initial gene expression matrix. In addition, we compared the reconstructed features with the original gene features to optimize single-cell classification by minimizing the MSELoss.

(5) $$\hat{X}=\mathrm{Dec}\left(H_{\mathrm{out}}\right)$$

(6) $$L_{\mathrm{FR}}=\sum_{i=1}^{B}{\left(X_{i}-{\hat{X}}_{i}\right)}^{2}$$

where *B* denotes the number of cells trained per batch, $\hat{X}$ denotes the matrix of gene expression values after reconfiguration and Dec denotes a two-layer MLP as a decoder.

#### 2.5.4 Virtual adversarial training

In order to enhance the robustness of the model, we further introduce an improved virtual adversarial training module into the pre-training of the model. Virtual adversarial training is a semi-supervised learning model (Miyato *et al.*, 2019), mainly by adding noise to the model input data to make the model adaptive against noise disturbance. Minimizing the Kullback–Leibler Divergence between the model perturbed by noise and the original model so that the model remains robust to perturbations. The random perturbations conform to the normal distribution and the dimension size is the same as the feature matrix, which is superimposed on the original feature input to input the model for training. First, we iterated IP times to find the direction of noise interference. The Kullback–Leibler Divergence loss between the output and the model without virtual adversarial training is calculated and back-propagated into the model and the noise is regularized with each iteration. After IP iterations, the final noise size is recalculated to obtain the loss in counter training, which is added into loss with labeled data to update gradient parameters. We calculate the LDS (local distributional smoothness), the direction r of the noise perturbation, concerning and the loss of virtual adversarial training from the LDS:

(7) $$\mathrm{LDS}=D_{KL}\left[p\left(y|x,\theta \right),p\left(y|x+\lambda \cdot r_{\mathrm{vat}},\theta \right)\right]$$

(8) $$r_{\mathrm{vat}}=\underset{r;\left\|r\right\|2\leq \epsilon }{\mathrm{argmax}}D_{KL}\left[p\left(y|x\right),p\left(y|x+r,\theta \right)\right]$$

(9) $$L_{\mathrm{VAT}}=\frac{1}{B}L\mathrm{DS}\left(x,\theta \right)$$

where $p\left(y|x,\theta \right)$ denotes the label prediction distribution *y* for input data *x* when the parameter vector is θ, $r_{\mathrm{vat}}$ denotes the direction of the perturbation of virtual adversary with λ as the weight parameter to balance the size of the perturbation, r is the randomly generated noise of the same size as the input data, $D_{\mathrm{KL}}$ measures the Kullback–Leibler divergence between the two distributions, and B denotes the batch size per training.

#### 2.5.5 Loss functions

Our model first receives the cross-entropy loss from the sample labels and the predicted labels, as well as the reconstructed expression value matrix loss from the decoder output during the training phase. The cross-entropy loss between the real and predicted results can be expressed as:

(10) $$L_{\text{FNN}}=-\sum_{i=1}^{B}\sum_{j=1}^{C}y_{i,\;j}\;\text{log}\;{\hat{y}}_{i,\;j},$$

where C denotes the number of cell classes in the sample, B denotes the batch size for training, $y_{i,\;j}$ is the true label in the sample, and ${\hat{y}}_{i,\;j}$ is the predicted label for model training.

Then after training on the input noise, the loss of the model to small noise perturbations which is the combination of three modular losses is added as the total loss to be minimized in training phase:

(11) $$L=L_{\mathrm{FNN}}+\lambda_{1}L_{\mathrm{FR}}+\lambda_{2}L_{\mathrm{VAT}},$$

where $L_{\mathrm{FNN}}$ represents the classification loss of the hybrid neural network, $L_{\mathrm{FR}}$ represents the feature reconstruction loss of gene expression, and $L_{\mathrm{VAT}}$ represents the virtual adversarial training loss. Parameters $\lambda_{1}$ and $\lambda_{2}$ are the weight of corresponding loss. We tested and obtained a balance between the three losses to update the optimal combination of parameters where $\lambda_{1}$ is set to 1 and $\lambda_{2}$ is set to 0.1.

## 3 Results

### 3.1 Implementation details and evaluation metrics

We implemented the HNNVAT model using PyTorch framework. The Xavier is adopted to initialize the model’s parameters and SGD is chosen to optimize the model. For the model configuration, we added four convolutional layers to the model and set the size of the convolutional kernel to 5. For the NN module, the number of neurons in hidden layer was set to 256, and the number of neurons in the output layer was set to 32. For training, we set the overall learning rate to 0.01 and set L2 regularization to 5e–4 in training phase to prevent overfitting. Meanwhile, to make the training faster, we adopt decay learning strategy, which requires only 100 epochs to obtain high accuracy. HNNVAT set the training batch size to 128 and output the test results after every 10 epochs to prevent overfitting of the model. Please refer to Supplementary Tables S1 and S2 for more information on network structure and parameter selection.

We divide the dataset into a training set, a validation set and a test set to guarantee the model’s performance during training. Eighty percent of the dataset is used to train HNNVAT, and the remaining average of 10% is used to test and validate the model results. In addition, we use Accuracy, Precision, Recall and F1-score to comprehensively evaluate the performance of the model, and plot receiver operating characteristic curves in two ways to demonstrate the excellent performance of HNNVAT.

### 3.2 Benchmarking classification methods

To further demonstrate the excellent performance of HNNVAT, we compared it with four advanced single-cell classification tools: scPred, CaSTLe, singleR and ACTINN. In addition, we compared it with a two-layer FNN classifier, the excellent single-cell classification model SigGCN and some traditional supervised classifiers, including RF, SVM-linear and KNN. The best values from all datasets are chosen for comparison in the aforementioned models, which use the same evaluation metrics as HNNVAT. These traditional classifiers are implemented using the scikit-learn package in Python.

### 3.3 Performance evaluation

#### 3.3.1 Comparison of HNNVAT with other related models

**Fig. 3.**
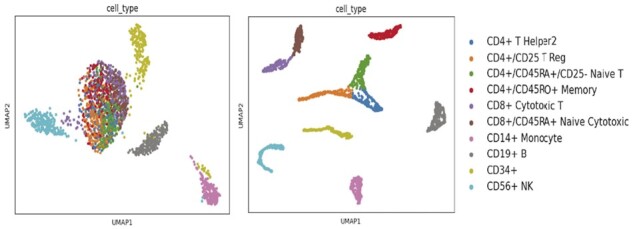
Visualization of the Zhengsorted test set by UMAP (uniform manifold approximation and projection) before and after HNNVAT classification

To demonstrate the superior generalization ability of HNNVAT on scRNA-seq data, we compared the experimental results of HNNVAT on the validation set for all datasets with benchmarking classification methods and the comparison results are summarized in Table 2. It can be observed that our proposed HNNVAT significantly outperforms other benchmarking classification methods including BaronMouse, BaronHuman, Muraro, Zhengsorted and Zheng68K on five of the six scRNA-seq datasets. Our HNNVAT outperformed the second-ranked sigGCN by 0.4% and 0.1% for the large single-cell datasets Zhengsorted and Zheng68K with more than 20 000 cells, respectively. And outperformed the third-place model by 3.3% and 1.6%, indicating that HNNVAT consistently outperformed the other methods in the case of large and complex data volumes. The HNNVAT model performs on par with the sigGCN model on the Segerstolpe dataset but outperforms the other methods on the remaining smaller datasets. The above observations show that our HNNVAT model consistently achieves the best or near-best performance on all datasets, especially for a large number of cells and complex datasets. The stable and superior performance of HNNVAT is attributed to the integrated feature lifting of the hybrid neural network and the enhanced model generalization performance of virtual adversarial training. Finally, we performed UMAP visualization on the test sets of all the datasets before and after classification by the HNNVAT model (see Supplementary Figs S6 and S7). As shown in Figure 3, the test set’s cell clusters of the Zhengsorted dataset can be distinguished clearly using HNNVAT.

**Table 2.** Validation set classification accuracy of the HNNVAT and the benchmarking classifiers on the six datasets

Classifier	BaronHuman cost	BaronMouse	Muraro	Segerstolpe	Zhengsorted	Zheng68K
HNNVAT	**0.982**	**0.989**	**0.995**	**0.977**	**0.926**	**0.753**
scPred	0.86	0.862	0.915	0.827	0.515	0.140
CaSTLe	0.971	0.91	0.972	0.953	0.836	0.736
singleR	0.951	0.868	0.977	0.953	0.723	0.388
ACTINN	0.977	0.984	0.991	0.958	0.845	0.737
FNN	0.967	0.958	0.986	0.967	0.893	0.668
sigGCN	0.979	0.974	0.991	**0.977**	0.922	0.752
RF	0.968	0.968	0.981	0.967	0.835	0.690
SVM-linear	0.824	0.704	0.981	0.383	0.859	0.652
KNN	0.953	0.926	0.991	0.935	0.825	0.594

Bold values denote the best performance for each column.

#### 3.3.2 Confusion matrix and ROC analysis

**Fig. 4.**
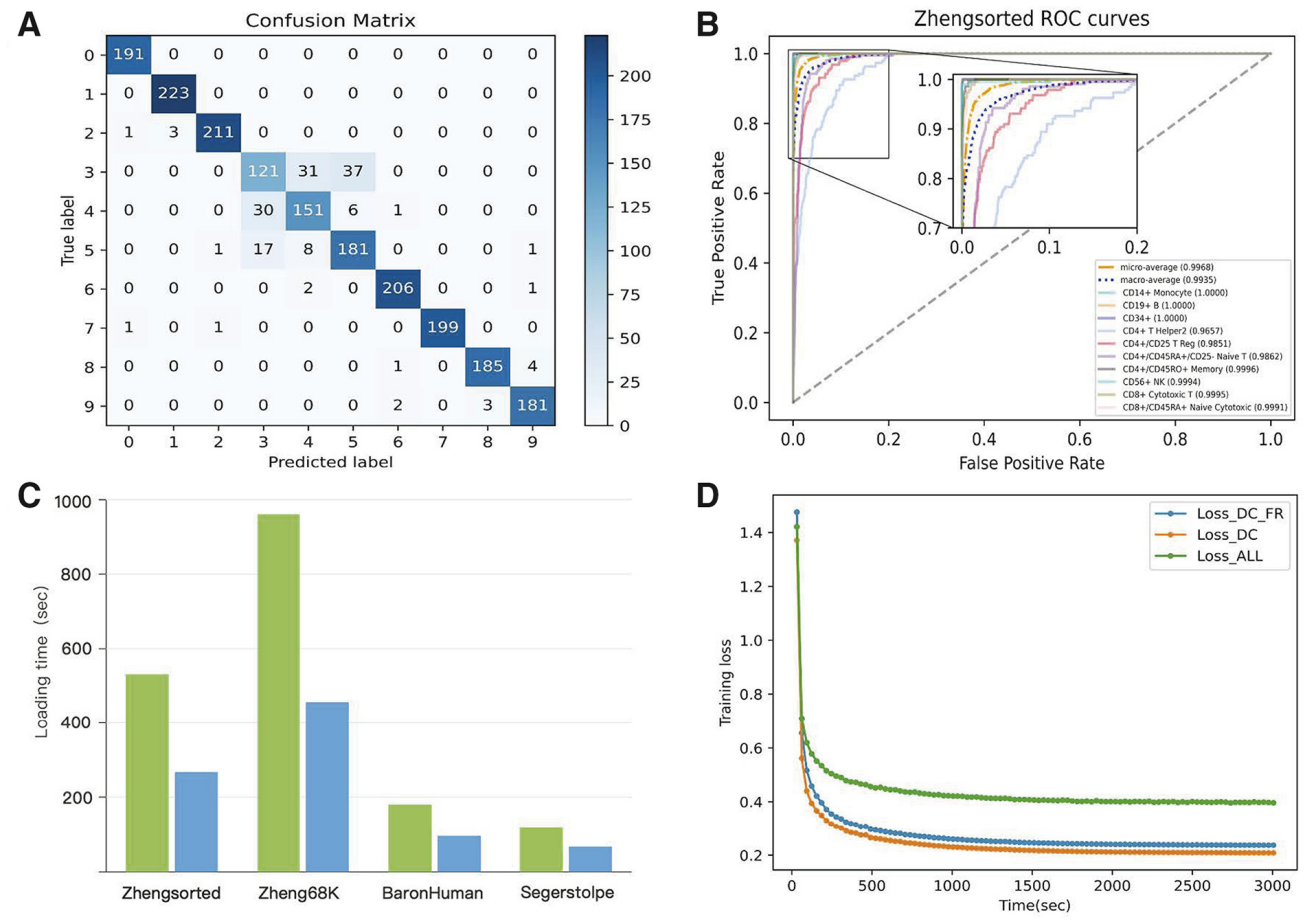
Presentation and ablation analysis of HNNVAT model classification results on the Zhengsorted dataset. (**A**) Confusion matrix of the prediction performance heatmap. (**B**) Average AUC and ROC curves using the 10 cell types. (**C**) Comparison of loading time before and after gene filtering. (**D**) Comparison of loss function convergence curves of different HNNVAT modules

We selected the ZhengSorted dataset to further validate the classification performance of HNNVAT since this dataset is more realistic with cells greater than 20 000. As shown in Figure 4A, we provided the confusion matrix of 10 categories. From Figure 4B, we could conclude that most of the categories obtain excellent results except for the confusion among the categories of CD4+ cells (the confusion matrixes of other datasets are shown in Supplementary Figs S1–S5), indicating CD4+ cells are not sufficiently heterogeneous. In addition, there usually exists an unbalanced distribution of cell classes in scRNA-seq dataset, and the distribution of positive and negative samples may be updated over time. Therefore, we performed ROC analysis on the results of each category predicted by HNNVAT (the Average AUC and ROC curves of other datasets are listed in Supplementary Figs S8–S12), and the overall AUC values were obtained by the mean AUC values of each category or reduction of prediction results to binary AUC values. The AUC values obtained by these two methods were 0.9974 and 0.9942, respectively, which indicates the model’s excellent classification performance.

### 3.4 Ablation experiments

To investigate the effectiveness of the main components of HNNVAT on the prediction results, we propose three variants on HNNVAT: (i) HNNVAT-NoVAT: HNNVAT with the virtual adversarial training module removed. (ii) HNNVAT-NoDC: feature extraction directly using only convolutional networks instead of extracting features through a dense connectivity mechanism and self-attention mechanism for aggregation. (iii) HNNVAT-NoFR: HNNVAT with the feature reconstruction module based on the encoder–decoder mechanism removed. The prediction results of the three HNNVAT variants are summarized in Table 3. It can be observed that the classification effect of HNNVAT-NoDC is not excellent enough, extracting only relatively simple fixed-dimension features through convolutional networks without the organic combination of features of different dimensions. After using a network structure combining dense connectivity mechanism and self-attention aggregation, the prediction effect is improved by at least 2.1%, which proves the effectiveness of hybrid neural networks. The performance of HNNVAT based on virtual adversarial training has improved significantly, proving that virtual adversarial training can help HNNVAT improve its generalization ability and also improve its robustness because of the input of noisy interference data. For the variant HNNVAT-NoFR without feature reconstruction, its classification ability on all datasets also leads to different degrees of degradation, and it can be shown that adding the loss of feature reconstruction to HNNVAT is positively helpful to assist in the global cell classification goal. The ablation experiments on HNNVAT further demonstrate the necessity of incorporating hybrid neural networks and virtual adversarial training modules to explore single-cell type assignment models.

**Table 3.** Prediction results of HNNVAT and its variants on the Zhengsorted dataset

Model	Accuracy	Precision	Recall	F1-score
HNNVAT	**0.926**	**0.926**	**0.923**	**0.920**
HNNVAT-NoVAT	0.910	0.908	0.910	0.912
HNNVAT-NoDC	0.905	0.905	0.910	0.903
HNNVAT-NoFR	0.918	0.919	0.915	0.915

Bold values denote the best performance for each column.

### 3.5 Running time evaluation

To verify the effectiveness of gene filtering, we recorded the model loading and its running time for one epoch of each benchmark dataset. Figure 4C shows the running time comparison before and after gene filtering for several datasets with larger data volumes. Although we only filtered out genes with expression values <5, dimensional transformation and ANOVA will be performed subsequently, and the model loading and running time are saved by a factor of two.

Finally, to compare the run efficiency of the main modules of HNNVAT, we compared loss values of different modules of HNNVAT on Zhengsorted dataset. Specifically, we performed 100 epochs and plotted loss curves of different modules according to their training times. As can be observed from Figure 4D, the loss values of different modules are not the same, mainly because the additive terms of local feature reconstruction and virtual adversarial training take up more computational costs. And the loss value of virtual adversarial training is usually larger than the value of neural network classification cross-entropy. However, these modules have similar loss convergence speeds, and all of them can make the model loss stabilize in a short time.

## 4 Discussion

Classification of single-cell by scRNA-seq datasets has become an essential step in the field of biology to explore cellular functions and associations of many complex diseases. In this article, we propose a supervised model that identifies cell types by feature expression relationships between genes and cells. HNNVAT uses a hybrid neural network structure with a dense connectivity mechanism and hierarchical attention aggregation. And node feature reconstruction is used to assist in the task of single-cell classification. Also, to prevent model overfitting, virtual adversarial training is used to enhance the generalization and stability of the model. Our benchmark experiments show that HNNVAT achieves excellent classification capability in six publicly available scRNA-seq datasets. In future studies, we will explore cellular and gene expression data about the interconnections between different biological tissues to increase multiple source information for cellular analysis.

## Supplementary Material

btad043_Supplementary_Data
