# Supplementary material for: Adversarial dense graph convolutional networks for single-cell classification
Source: Bioinformatics. 2023 Jan 20;39(2):btad043. doi: 10.1093/bioinformatics/btad043 (PMC9919433; doi:10.1093/bioinformatics/btad043)
Supplement: btad043_Supplementary_Data [file btad043_supplementary_data.pdf]

Supplementary Material:

Adversarial dense graph convolutional networks for single-cell classification

Kangwei Wang [1], Zhengwei Li [1,*], Zhu-Hong You [2,*], Pengyong Han [3,*] and Ru Nie [1]

[1]School of Computer Science and Technology, China University of Mining and Technology, Xuzhou, 221116, China, [2]School of Computer Science, Northwestern Polytechnical University, Xi'an 710072, China and [3]Central Lab, Changzhi Medical College, Changzhi 046000, China

*To whom correspondence should be addressed.

The supplementary file includes:

1. Supplementary Tables

Table S1. The hyper-parameter search ranges and the optimized values of HNNVAT.

Table S2. The overall network structure parameters of HNNVAT.

2. Supplementary Figures

Fig. S1 - S5. Confusion matrix of the prediction performance heatmap comparison of category analysis for scRNA-seq dataset on the HNNVAT model.

Fig. S6 - S7. The visualization of the Zhengsorted test set by UMAP (uniform manifold approximation and projection) before and after HNNVAT classification.

Fig. S8 - S12. Average AUC and ROC curves comparison of category analysis for scRNA-seq dataset on the HNNVAT model.

1. Supplementary Tables

Table S1. The hyper-parameter search ranges and the optimized values of HNNVAT.

Modules	Parameters	Search range	Final
Feature reconstruction	Loss weight1	[0.5, 0.8, 1, 1.5, 2]	1
Neural network	Layers of GCN	[3, 4, 5, 6, 7]	4
	Dropout rate	[0.1, 0.3, 0.5, 0.7]	0.3
	Hidden size	[64, 128,256]	128
Virtual adversarial training	Loss weight2	[0.01, 0.1, 0.2, 0.5, 1]	0.1
Other parameters	Learning rate	[0.1, 0.01, 0.001]	0.01

Table S2. The overall network structure parameters of HNNVAT.

Modules		Parameters
Dense GCNS		a) GCN (Convolution kernels = 5)<br>b) Attention layer1(in_features = 1000, out_features = 64, bias = True)<br>c) Attention layer2(in_features = 64, out_features = 1, bias = True)<br>d) ReLU ()
Max pooling		a) MaxPooling (poolsize=8)<br>b) Flatten ()
Fully connected layers	Linear 1	a) Linear (in_features = 1000, out_features = 128, bias = True)<br>b) ReLU ()
	Linear 2	a) Linear (in_features = 128, out_features = 32, bias = True)<br>b) ReLU ()
Output layers		a) Linear (in_features = 64, out_features = Number of cell types, bias = True)<br>b) Softmax()
Feature reconstruction		a) Linear (in_features = 32, out_features = 1000, bias = True)<br>b) ReLU ()

2. Supplementary Figures

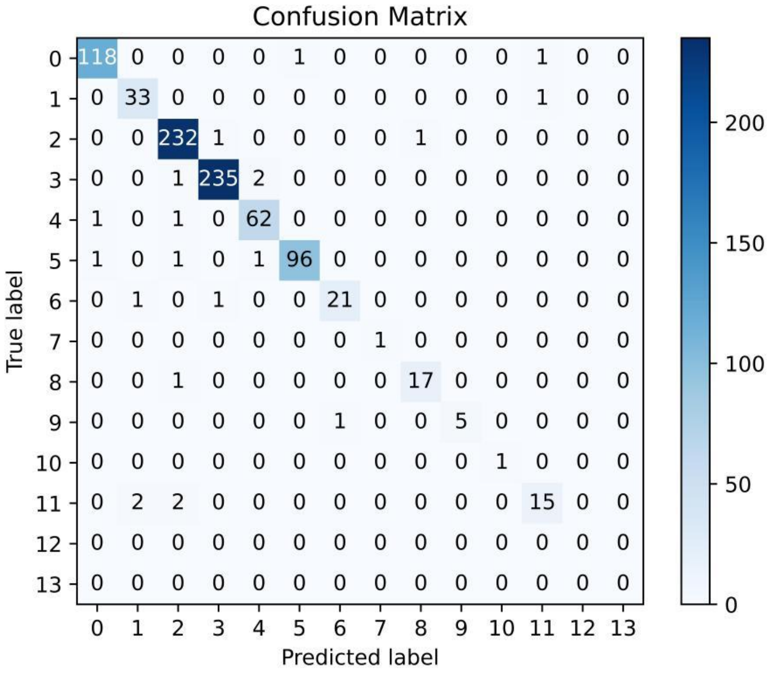

Fig. S1. Confusion matrix of the prediction performance heatmap comparison of category analysis for BaronHuman dataset on the HNNVAT model.

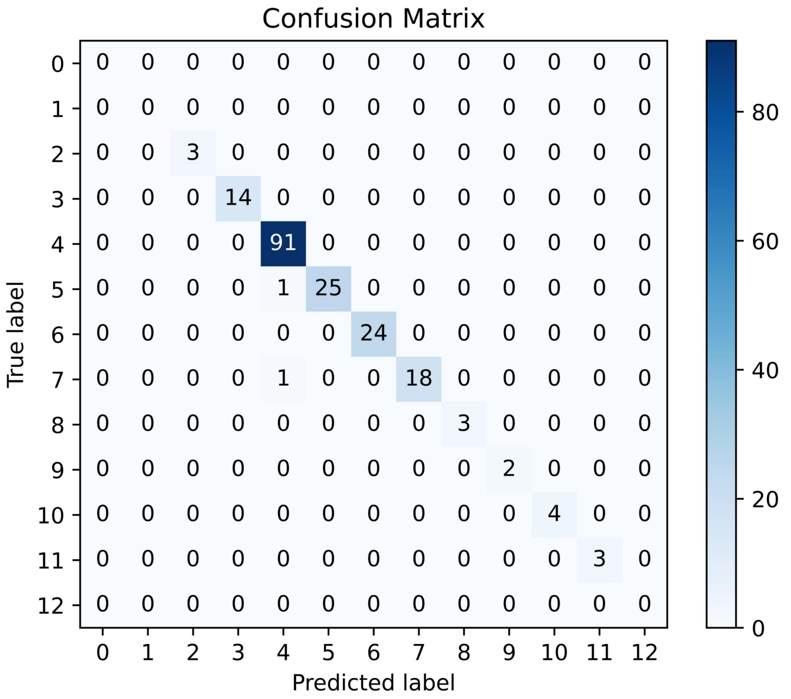

Fig. S2. Confusion matrix of the prediction performance heatmap comparison of category analysis for BaronMouse dataset on the HNNVAT model.

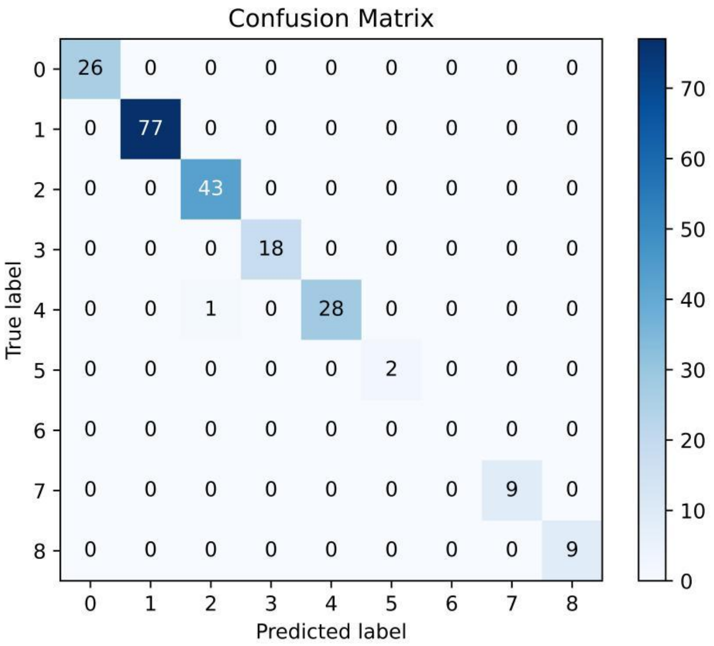

Fig. S3. Confusion matrix of the prediction performance heatmap comparison of category analysis for Muraro dataset on the HNNVAT model.

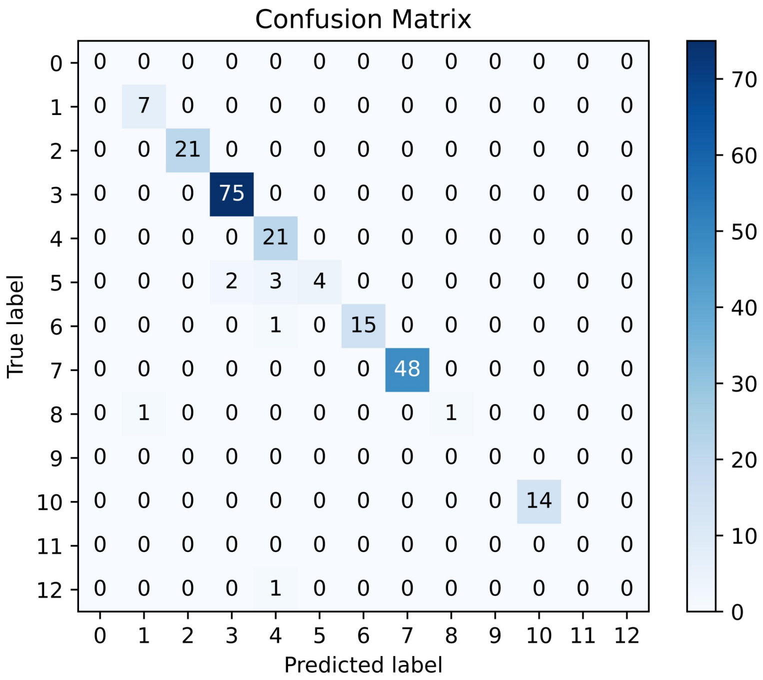

Fig. S4. Confusion matrix of the prediction performance heatmap comparison of category analysis for Segerstolpe dataset on the HNNVAT model.

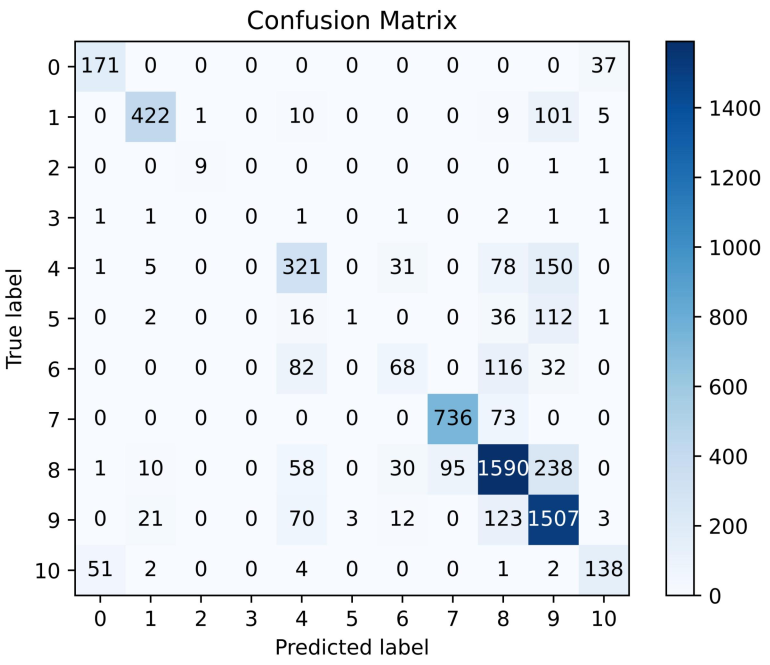

Fig. S5. Confusion matrix of the prediction performance heatmap comparison of category analysis for Zheng68K dataset on the HNNVAT model.

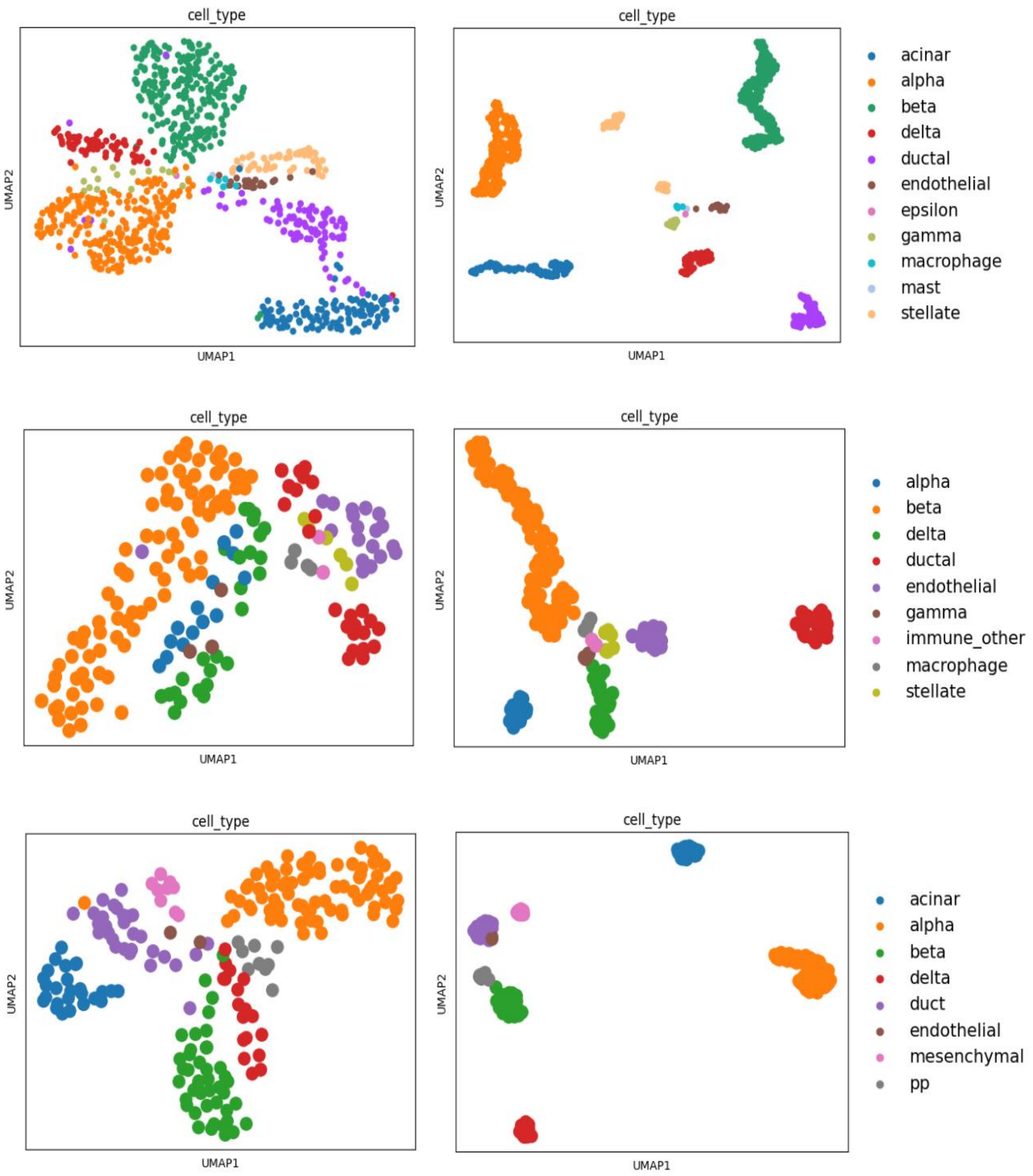

Fig. S6. The visualization of the Zhengsorted test set by UMAP (uniform manifold approximation and projection) before and after HNNVAT classification. On the left is the test set after data preprocessing, and on the right is the result after model classification. The data sets from the top down are BaronMouse, BaronHuman and Muraro.

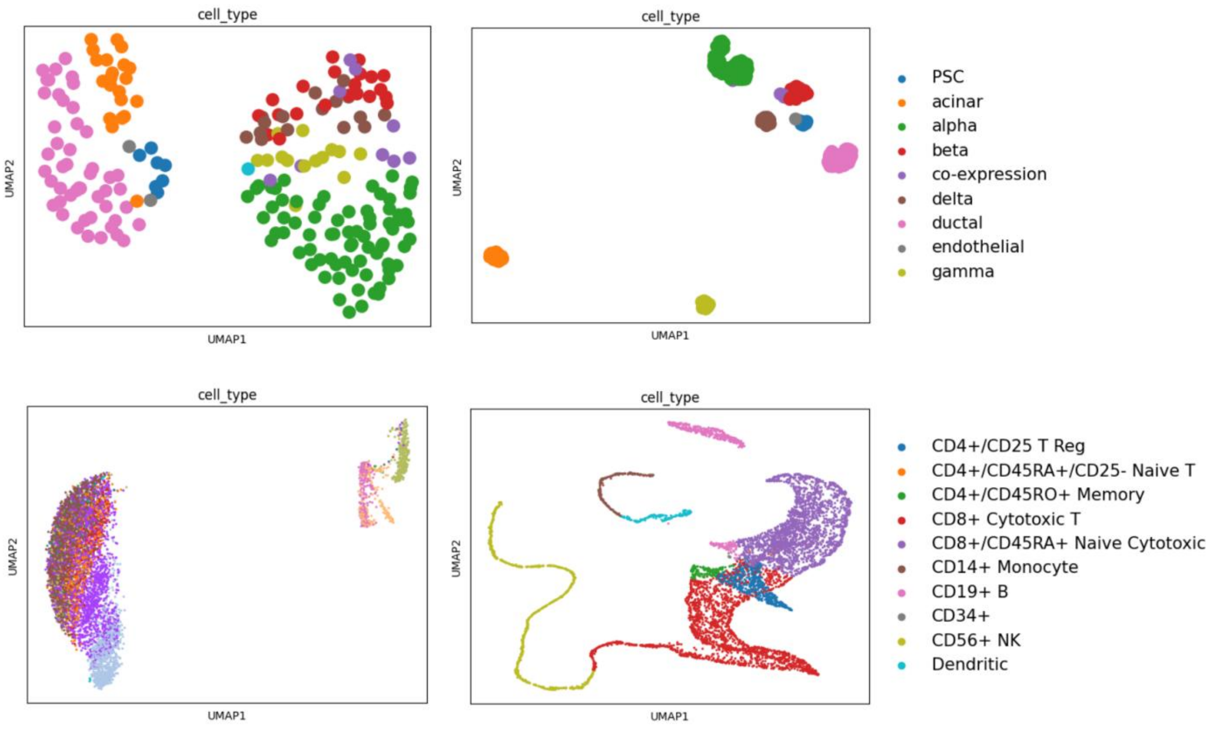

Fig. S7. The visualization of the Zhengsorted test set by UMAP (uniform manifold approximation and projection) before and after HNNVAT classification. On the left is the test set after data preprocessing, and on the right is the result after model classification. The top test set is Segerstolpe, and the bottom test set is Zheng68K.

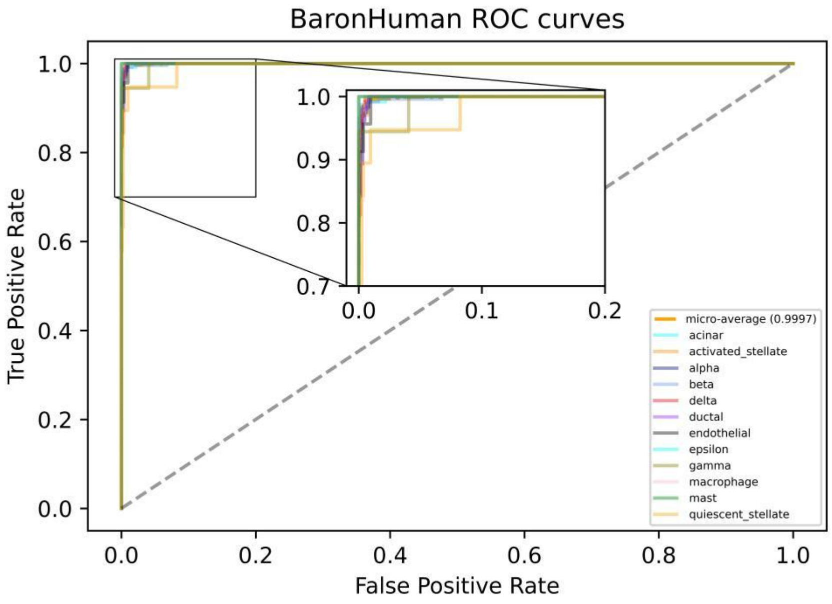

Fig. S8. Average AUC and ROC curves comparison of category analysis for BaronHuman dataset on the HNNVAT model.

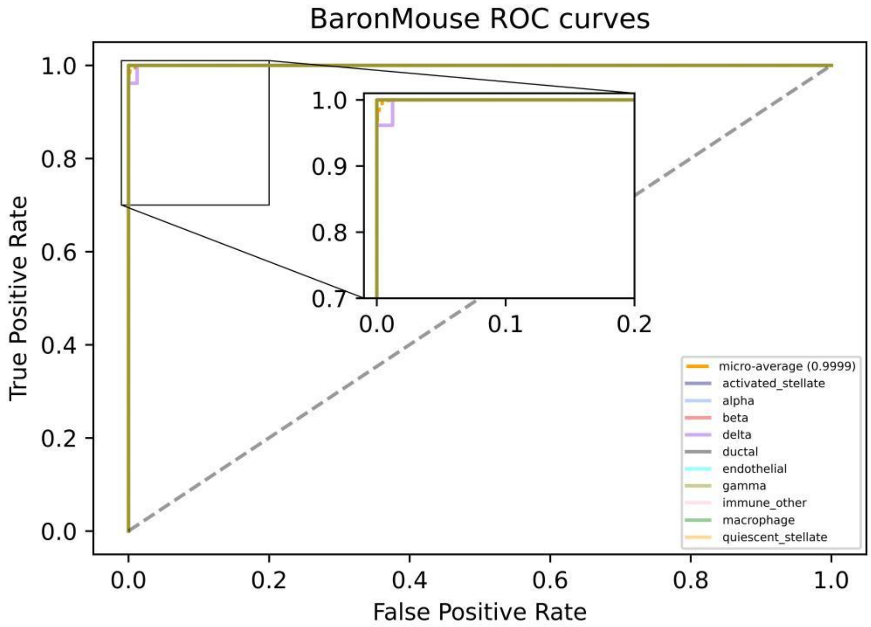

Fig. S9. Average AUC and ROC curves comparison of category analysis for BaronMouse dataset on the HNNVAT model.

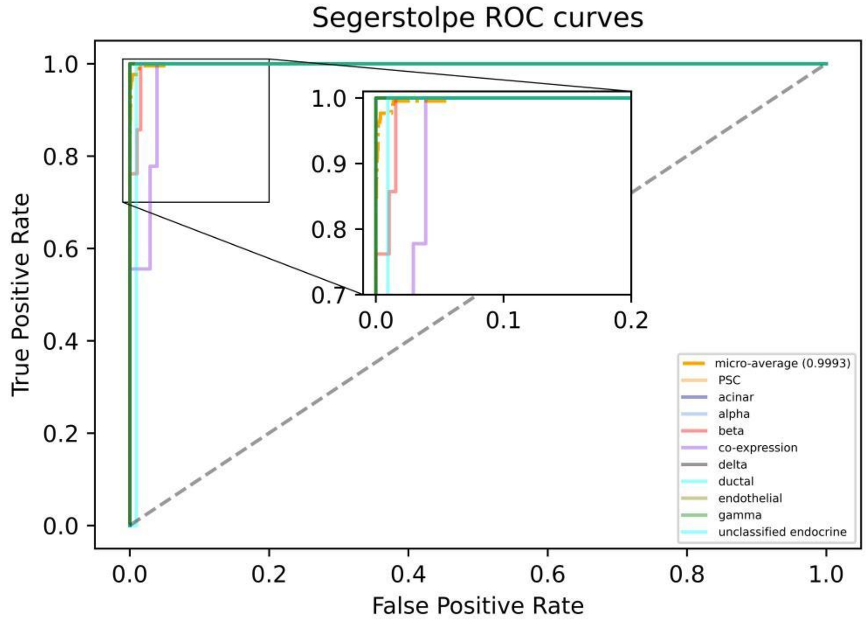

Fig. S10. Average AUC and ROC curves comparison of category analysis for Muraro dataset on the HNNVAT model.

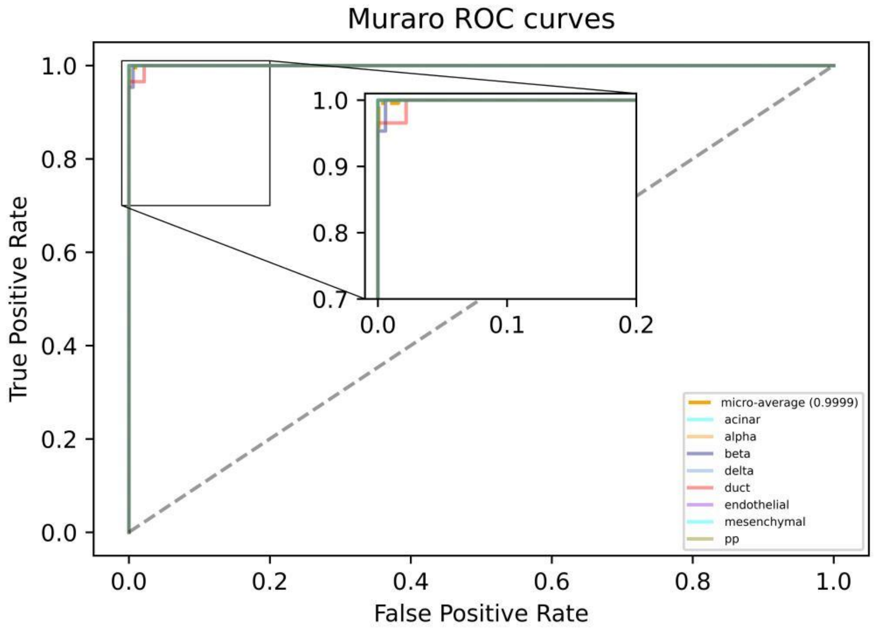

Fig. S11. Average AUC and ROC curves comparison of category analysis for Segerstolpe dataset on the HNNVAT model.

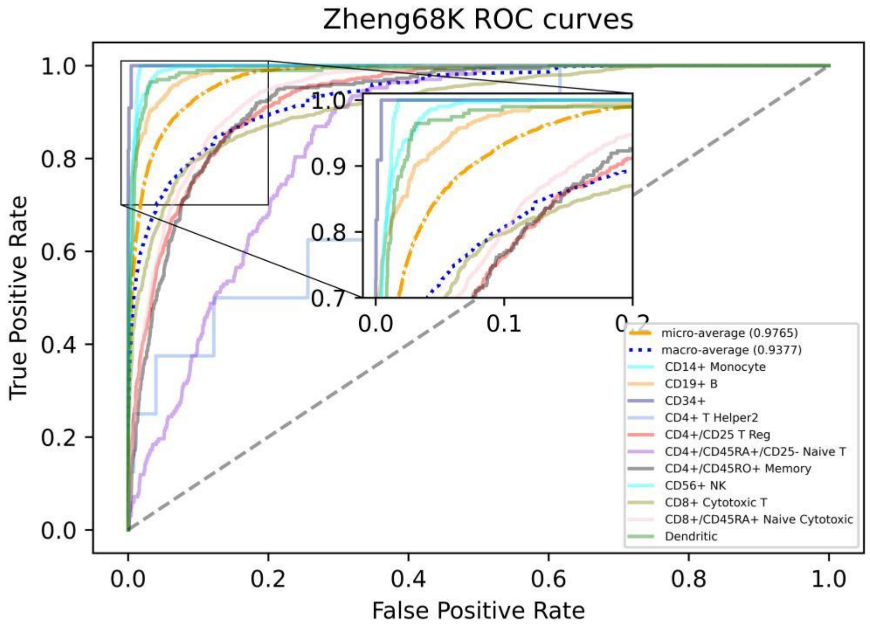

Fig. S12. Average AUC and ROC curves comparison of category analysis for Zheng68K dataset on the HNNVAT model.
